# Prevalence of anxiety, depression, and stress in patients with multiple sclerosis in Kermanshah-Iran: a cross-sectional study

**DOI:** 10.1186/s12888-020-02579-z

**Published:** 2020-04-15

**Authors:** Saba Karimi, Bahare Andayeshgar, Alireza Khatony

**Affiliations:** 1 MSc. in Medical Surgical Nursing, Clinical Research Development Center, Imam Reza Hospital, Kermanshah University of Medical Sciences, Kermanshah, Iran; 2 MSc. in Statistics, Clinical Research Development Center, Imam Reza Hospital, Kermanshah University of Medical Sciences, Kermanshah, Iran; 3 Health Institute, Social Development and Health Promotion Research Center, Kermanshah University of Medical Sciences, Kermanshah, Iran; 4 Clinical Research Development Center, Imam Reza Hospital, Kermanshah University of Medical Sciences, Kermanshah, Iran

**Keywords:** Anxiety, Depression, Multiple sclerosis, Prevalence, Stress

## Abstract

**Background:**

Multiple sclerosis (MS) is a chronic disease that decreases the physical ability and affects the mental health of the patients. This descriptive-analytical study investigated the prevalence of depression, anxiety and stress in MS patients.

**Methods:**

A total of 87 MS patients were recruited in this study through simple random sampling method using a random number table. Data were collected by Depression, Anxiety, and Stress Scale-21 (DASS-21) and analyzed by descriptive and analytical statistics.

**Results:**

The mean age of the patients was 35.5 ± 9.2 years. Of them, 41 (47.1%) had moderate depression, 34 (39.1%) had moderate anxiety, and 39 (44.8%) had moderate stress. There was a significant relationship between depression and job, education, and economic status of the participants. There was also a significant association between the participants’ economic status and anxiety. There was no significant relationship between stress and any of the variables.

**Conclusion:**

Given the relatively high prevalence of anxiety, depression, and stress in MS patients as well as the significant relationship between their economic status and depression and anxiety, interventional measures are required to be taken to decrease their problems and to provide a favorable ground for their employment. Periodic examinations by psychologists / psychiatrists and treatment of patients with symptoms of stress, anxiety and depression are also essential.

## Background

Multiple sclerosis (MS) is one of the debilitating diseases of the central nervous system that is mostly diagnosed in the age range of 20–40 years. It is characterized by inflammation, shrinkage of the sheath of neurons, and formation of plaque in different areas of the brain [1]. The prevalence of MS is 57–78 per 100,000 people. Approximately 2.5 million people have been estimated to suffer from this disease worldwide [2]. The number of MS patients in Iran is 60 per 100,000 people [3]. According to the latest statistics from the Ministry of Health and Medical Education for 2016, there are 60,000 patients with multiple sclerosis in Iran [4], 43.3% of whom belong to Kermanshah [3].

The etiology of this disease is not yet known(2) [2] but factors such as immune system deficiency, genetic predisposition, lack of vitamin D, Epstein-Barr virus, family background, geographical region, stress, and lifestyle play a role in this disease [5].

The increasing growth of MS in Iran in recent years makes it more important [5, 6]. Most MS patients are 20–40-year-old women who are at the peak of their physical, psychological, and social activities [1–3, 6]. One of the major problems of MS patients is psychological problems [7] as most of them suffer from anxiety, depression, and stress [8]. The words stress and anxiety are often used interchangeably but they are two different experiences. Anxiety is a feeling not directly related to external stimulus, but stress is a reaction that occurs in response to a specific external stimulus [9].

Evidence has shown that risk of depression, stress, and anxiety is higher in MS patients than in healthy people [10]. A study in Canada (2018) showed 30% of MS patients suffered from anxiety and 16.3% were affected with depression. Anxiety, depression, and stress are negative loads of MS that have negative effects on the quality of life of the patients [11]. Another study in Italy (2011) indicated 43.0% of MS patients suffered from anxiety and 34% suffered from depression [8]. The results of a study in the U.S (2017) revealed 20.6% of MS patients suffered from depression [12].

Hence, given the increasing prevalence of MS in Iran, shortage of information about the frequency of anxiety, depression, and stress in these patients, and the role of geographical region and lifestyle factors in the development of this disease, the current study was conducted to determine the prevalence of anxiety, depression, and stress in MS patients in Kermanshah, a western province of Iran.

## Methods

### Study design

This cross-sectional study was carried out over 4 months from 27.9.2016 to 27.1.2017 in Kermanshah, a western province of Iran, according to the STROBE guidelines. This study sought to determine the prevalence of depression, anxiety, and stress in MS patients.

### Study hypothesis

It was hypothesized that prevalence of stress, anxiety and depression in patients with MS is high.

### Sample

The study population comprised of 1025 MS patients under the coverage of the MS Society of Kermanshah. The study sample was computed to be 87 patients using Cochrane’s formula based on the study of Boeschoten (2017) [12], a confidence level of 95%, and the risk of loss of 15%. The participants were selected by random sampling method from the MS Society of Kermanshah. The inclusion criteria consisted of willingness to participate in the study, having an active medical file in the MS center, reading and writing ability, history of a minimum of 6 months of MS, having a stable clinical condition, absence of physical illnesses such as cancers, absence of mental illnesses (such as depression, schizophrenia, and bipolar disorders), not taking psychotropic drugs such as sedatives and opioids, ability of verbal communication in Persian, and absence of acute or chronic physical disorders (such as cardiovascular, respiratory, hepatic, musculoskeletal, and renal diseases).

### Instrument

An information form and Depression Anxiety and Stress Scale-21 (DASS-21)were used for data collection. The information form consists of 10 items on age, gender, education, job, monthly income, marital status, history of the disease, family history of MS, duration of disease, and physical activities. The education levels were divided by less than a high school diploma, a high school diploma, and an undergraduate college education.

DASS-21 is a standard and self-report tool developed by Antony et al. (1998) to measure the severity of a range of symptoms related to depression, anxiety and stress among individuals without a depression, anxiety and stress diagnosis [8]. Content validity was sued to measure the validity of this scale [13, 14]. Covice et al. (2012) studied the internal consistency of the questionnaire using Cronbach’s alpha and reported the reliability index of 0.94 for depression and 0.87 for anxiety [13]. Miller et al. (2006) also reported the Cronbach’s alpha levels of 0.92 and 0.81 for stress and anxiety, respectively [15]. The Persian version of DASS-21 has been psychometrized by Sahebi et al. (2005) in Iran and the Cronbach’s alpha levels of 0.7, 0.67, and 0.49 for depression, anxiety, and stress dimensions of DASS-21 were reported, respectively [14]. The Cronbach’s alpha levels indicated acceptable reliability of DASS-21.

DASS-21 has 21 items and three subscales of depression, anxiety, and stress. Each subscale includes 7 items. The depression subscale involves statements like poor mood, lack of self-confidence, despair, worthlessness of life, disinterest in participation in affairs, not enjoying life, and lack of energy. The anxiety subscale evaluates elements such as physiologic excitement, anxiety, and situational fear. The stress subscale involves statements such as patient’s comfort, patient’s reaction to different situations, distress and confusion, patience level, and irritability. Responses are rated based on the four-point Likert scale, including never, often, sometimes, and always, which are scored from 0 to 3. Since DASS-21 is the short form of the main scale, which includes 42 items, the final score of each subscale must be doubled. In the depression section, score 0–9 shows normal (absence of depression), 10–13 mild depression, 14–20 moderate depression, 21–27 severe depression, and > 27 very severe depression. In the anxiety section, score 0–7 shows normal (absence of anxiety), 8–9 mild anxiety, 10–14 moderate anxiety, 15–19 severe anxiety, and > 19 very severe anxiety. As for the stress section, score 0–14 indicates normal (absence of stress), 15–18 mild stress, 19–25 moderate stress, 26–33 severe stress, and > 33 very severe stress [13].

### Data collection

**Fig. 1:**
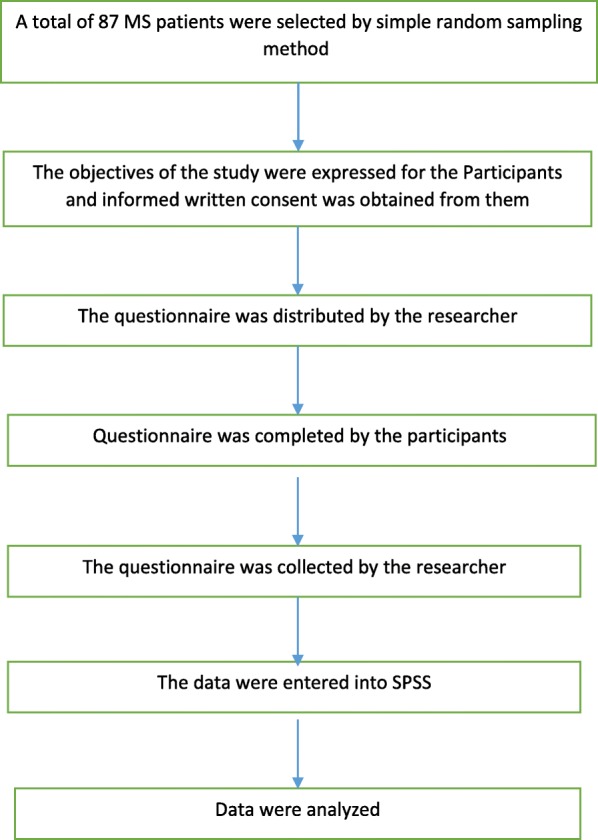
Participant flow diagram

For data gathering, the researcher referred to the MS center of Kermanshah and proceeded to choose the qualified participants. First, the study objectives were explained to the patients and their consent for participation in the study was taken. Then, the questionnaires were given to the participants and after compilation were collected by the researcher (Fig. 1).

### Data analysis

Data were analyzed by the Statistical Package for Social Sciences (SPSS v.18.0; SPSS Inc., Chicago, IL, USA) using descriptive statistics (mean and standard deviation) and inferential statistics (ANOVA, independent t-test, Pearson product moment correlation test, and multivariate linear regression). In terms of education, the participants were subdivided into three categories: under diploma, diploma, and college education. In terms of economic status, the participants were classified into one of the low-income (≤$238), middle-income ($239–$714), and good-income (≥$715) classes, based on their monthly income. In this regard it should be noted that monthly income of less than $540 in Iran means living below the poverty line, and the average salary in Iran is $220 per month [16]. To analyze the data, the distribution of stress, anxiety, and depression variables was analyzed by the Kolmogorov-Smirnov test, which showed they were normally distributed. Then, the Pearson product moment correlation test was run to study the association between stress, anxiety, and depression variables and independent variables (economic status and education). The Pearson product moment correlation test was also used to analyze the correlation between age and stress, anxiety, and depression variables. To better understand the results, age was grouped into four categories. The multivariate regression model was applied to analyze the concurrent relationship between demographic variables and stress, anxiety, and depression. Since the variables job, education, and economic status were correlated with depression, the linear regression model was applied to moderate their interaction. The mentioned variables were included in the model using the Enter method. The level of significance was set at *p* < 0.05.

### Ethical considerations

The Ethical Review Committee of Kermanshah University of Medical Sciences approved the study (no: KUMS.REC.1395.112). The study objectives were explained to all participants and they were assured their personal data and responses would be kept confidential. Informed written consent was taken from all participants.

## Results

In this research, 87 MS patients were studied. Their mean age was 35.5 ± 9.2 years, with an age range of 20–62 years. The mean duration of the disease diagnosis was 6.2 ± 5.7 years. Most of the participants (70.1%, *n* = 61) were female. Further, 54 (62.1%) patients were married, 35 (40.2%) had high school diploma, 49 (56.3%) were homemaker, and 11 (12.6%) had a family history of MS. About half of them (52.9%, *n* = 46) had a monthly income of 239-$714. The minimum and maximum periods of disease diagnosis were 3 and 31 years, respectively. Moreover, 40 (46.0%) of them had 56.0 ± 30.4 min daily physical activity. The minimum and maximum physical activity periods were 20 and 120 min, respectively (Table 1). The results showed 21 (24.1%) patients had severe depression and 41 (47.1%) had moderate depression (Table 2). The patients with an age range of 30–40 years (9.4 ± 5.1) and those aged > 50 years (6.5 ± 4.8) had the highest and lowest levels of depression, respectively. With respect to sex, the mean levels of depression in the females and males were 8.4 ± 5.1 and 7.5 ± 4.2, respectively, indicating no significant difference between them. As for marital status, the maximum and minimum levels of depression were found in the married (8.6 ± 5.1) and widowed patients (6.5 ± 3.5), respectively, which showed no significant difference (Table 3).

**Table 1:** Demographic characteristics of participants

Variables		Number (%)
Gender	Male	26(29.9)
	Female	61(70.1)
Age (years)	20–29	28(32.2)
	30–39	34(39.1)
	40–49	19(21.8)
	≥50	6(6.9)
Marital status	Single	31(35.6)
	Married	54(62.1)
	Divorced and aligned	2(2.3)
Education	Less than a high school diploma	27(31.0)
	High school diploma	35(40.2)
	Undergraduate college education	25(28.7)
Occupation	Housekeeper	49(56.3)
	Unemployed	17(19.5)
	Self-employed	13(14.9)
	Employee	5(5.7)
	Retired	3(3.4)
Economic status (monthly income in dollar)	Low (≤238)	26(29.9)
	Moderate (239–714)	46(52.9)
	Good (≥715)	15(17.2)
History of the disease	Yes	24(27.6)
	No	63(72.4)
Family history	Yes	11(12.6)
	No	76(87.4)
Smoking	Yes	7(8.0)
	No	80(92)
Physical activity	Yes	40(46.0)
	No	47(54.0)

**Table 2:** Frequency distribution of depression, anxiety and stress in participants

Variables	Categories	Number (%)
Depression	Normal (absence of depression) (0–9)	25(28.7)
	Moderate (10–13)	41(47.1)
	Sever (14–20)	21(24.1)
Anxiety	Normal (absence of anxiety) (0–7)	23(26.4)
	Moderate (8–9)	34(39.1)
	Sever (10–14)	30(34.5)
Stress	Normal (absence of stress) (0–14)	28(32.2)
	Moderate (15–18)	39(44.8)
	Sever (19–25)	20(23.0)

**Table 3:** Relationship between demographic variables and depression, anxiety and stress

Variables		Depression Mean ± SD^a^	*p*-value	Anxiety Mean ± SD	*p*-value	Stress Mean ± SD	*p*-value
Gender	Male	7.5 ± 4.2	0.413	9.9 ± 5.7	0.347	6.9 ± 4.7	0.216
	Female	8.4 ± 5.1		8.6 ± 5.8		8.4 ± 5.5	
Family history	Yes	8.2 ± 3.8	0.985	10.0 ± 5.9	0.553	7.2 ± 5.6	0.877
	No	8.1 ± 5.0		8.9 ± 5.8		7.4 ± 4.9	
History of the disease	Yes	8.7 ± 4.8	0.554	9.1 ± 5.9	0.985	7.4 ± 5.7	0.949
	No	7.9 ± 4.9		9.0 ± 5.8		7.4 ± 4.8	
Smoking	Yes	6.4 ± 2.6	0.136	8.7 ± 5.4	0.884	7.3 ± 3.8	0.949
	No	8.3 ± 5.0		9.0 ± 5.9		7.4 ± 5.1	
physical activity	Yes	7.5 ± 4.8	0.284	8.7 ± 5.6	0.680	6.8 ± 5.0	0.111
	No	8.6 ± 4.8		9.2 ± 5.9		8.2 ± 4.9	
Marital status	Single	7.5 ± 4.6	0.531	9.6 ± 5.6	0.751	6.8 ± 4.7	0.221
	Married	8.6 ± 5.1		8.70 ± 5.9		7.9 ± 5.2	
	Divorced and aligned	6.5 ± 3.5		8.0 ± 9.9		2.5 ± 2.1	
Occupation	Housekeeper	9.1 ± 5.1	0.023	8.9 ± 6.1	0.154	7.4 ± 5.0	0.601
	Unemployed	7.0 ± 3.4		10.1 ± 5.3		7.7 ± 5.4	
	Self-employed	6.9 ± 4.8		8.1 ± 5.6		7.0 ± 5.2	
	Employee	3.2 ± 3.1		4.6 ± 2.7		5.4 ± 4.3	
	Retired	12.3 ± 4.7		14.7 ± 5.1		11.3 ± 2.5	

^a^Standard deviation

Regarding the economic status, the findings showed the patients with a monthly income of $715 had the minimum level of depression (8.2 ± 4.9). The maximum level of depression was reported for the patients with a monthly income of $239–$714 (8.9 ± 5.4). The results of the Spearman correlation coefficient test showed a significantly reverse correlation between depression and economic status (*r* = − 0.268, *p* = 0.012) and education (*r* = − 0.297, *p* = 0.009) (Table 4). With regard to the job, the retired patients (12.3 ± 4.7) and employees (3.2 ± 3.1) had the maximum and minimum levels of depression, respectively, indicating a significant difference (*p* = 0.023). In terms of education, patients with academic education (6.04 ± 6.06) and those with primary education (9.6 ± 4.8) had the minimum and maximum levels of depression, respectively. As for physical activity, the patients with physical activity (7.5 ± 4.9) had lower depression than those without physical activity (8.7 ± 4.8), which showed no significant difference (Table 3). The results of Pearson correlation coefficient test indicated that the patients’ depression increased with a rise in their age (*r* = 0.66), but it showed no statistically significant correlation (Table 4).

**Table 4:** Relationship between anxiety, depression, and stress with independent ratings variables

Variables	Test result	Economic Status	Education	Age
Anxiety	Correlation Coefficient	−0.29	−0.1	0.060
	*p*-value	0.006	0.355	0.550
Depression	Correlation Coefficient	−0.27	− 0.28	0.660
	*p*-value	0.012	0.009	0.540
Stress	Correlation Coefficient	−0.16	−0.14	0.04
	*p*-value	0.141	0.190	0.689

The findings showed 30 (34.5%) participants had severe anxiety and 34 (39.1%) had moderate anxiety (Table 2).

The participants in the age ranges 30–39 (9.9 ± 5.9) and 40–49 (7.5 ± 6.4) had the highest and lowest levels of anxiety, respectively. Regarding gender, the mean anxiety was higher in men (9.9 ± 5.7) than in women (8.6 ± 5.8), indicating no statistically significant difference. As for marital status, the maximum and minimum levels of anxiety were found for the single (9.6 ± 5.6) and widowed (8.0 ± 9.9) patients, which showed no statistically significant difference. Regarding job, the retired patients (14.7 ± 5.1) and employees (4.6 ± 2.7) had the highest and lowest levels of anxiety, respectively, showing no statistically significant difference. (Table 4). In terms of economic status, the results showed that patients with monthly income above $715 had the lowest level of anxiety (5.5 ± 4.3). The highest level of anxiety was reported for the patients with monthly income below $238 (10.6 ± 5.7). There was a significantly reverse correlation between economic status and anxiety (*r* = − 0.291, *p* = 0.006) (Table 4). With respect to education, the results indicated the patients with primary education (9.9 ± 5.9) and those with academic education (8.5 ± 5.2) had the highest and lowest levels of anxiety, respectively, which showed no statistically significant difference. The results of physical activity revealed the patients with physical activity (8.7 ± 5.7) had lower anxiety than those without physical activity (9.2 ± 5.9), indicating no statistically significant difference (Table 3). Further, there was no significant association between age and anxiety (Table 4).

The findings showed 20 (23.0%) patients had severe stress and 39 (44.8%) had moderate stress (Table 2). Regarding gender, the mean stress was higher in women (8.4 ± 5.5) than in men (6.9 ± 4.7), which showed no statistically significant difference. In terms of marital status, the married (7.9 ± 5.2) and widowed patients (2.5 ± 2.1) had the highest and lowest levels of anxiety, respectively, showing no statistically significant difference (Table 3). As for the job, the retired patients (11.3 ± 2.5) and employees (5.4 ± 4.3) had the highest and lowest levels of stress, but the difference was not statistically significant. For physical activity, the patients with physical activity (6.5 ± 5.0) had less stress than those without physical activity (8.2 ± 4.9), indicating no significant difference (Table 3).

Patients in the age ranges of 30–39 (8.0 ± 5.2) and more than 50 years (6.7 ± 5.2) had the highest and lowest levels of stress, showing no statistically significant difference. With regard to the economic status, the highest level of stress was related to the patients with monthly income below $238 (8.6 ± 5.9) and the lowest level of stress was found for the patients with monthly income above $715 (5.8 ± 3.9). There was no significant correlation between stress and economic status (Table 4). As for education, the patients with junior high school education (8.6 ± 5.9) and those with academic education (6.7 ± 4.8) had the highest and lowest levels of stress, respectively, showing no significant difference (Table 4).

The results of the multivariate model indicated none of the independent variables (gender, family history of MS, history of neural diseases, smoking, physical activity, age, economic status, education, marital status, and job) had simultaneously significant effects on depression, anxiety, and stress. The variables job, education, and economic status were correlated with depression, so the linear regression model was used to moderate their interaction. The findings showed only job could predict depression among the three mentioned variables. Moreover, the odds of depression were significantly lower in employees than retired patients (OR = -0.387) (Table 5). In addition, the results of adjusted R-Squared showed 12% of depression changes could be explained by the job variable. The results also showed the fitted linear regression model was significant (*p* = 0.012).

**Table 5:** Multiple regression with ENTER method for variable of depression

	Beta	t	*p*-value
Constant		5.03	< 0.001
Education	−0.09	−0.81	0.420
Income	−0.20	−1.82	0.070
Occupation			
Housekeeper	−0.35	−1.24	0.220
Unemployed	−0.46	− 1.89	0.060
Self-employed	−0.37	−1.74	0.080
Employee	−0.39	−2.38	0.020

R square:0.18

Adjusted R square:0.12

## Discussion

The present study was aimed to determine the prevalence of depression, anxiety, and stress in MS patients. In the present study, about one-fourth of the patients suffered from depression and severe anxiety and one-third of them had severe anxiety. Dehghani et al. (2013) conducted a study on 110 MS patients in Tehran, Iran and reported 46.4% of them had severe stress, 19.2% had anxiety, and 29.2% had severe depression [17]. Zarei et al. (2013) also carried out a study on 60 MS patients in Zahedan, Iran and found 48.3, 53.3, and 58.3% of them had stress, anxiety, and depression, respectively [18]. Beiske et al. (2008) studied the prevalence of anxiety and depression in the MS patients and found 31.4% of patients had anxiety and 19.3% had depression [19]. The results of our study and those of the above study were similar with respect to the prevalence of anxiety and depression. The results of the study by Alsaadi et al. (2015) in Emirates showed from 80 MS patients, 17% had severe depression and 20% had moderate Anxiety. Moreover, no significant relationship was found between anxiety and depression and variables age, sex, and education [20]. Regarding the relatively high prevalence of anxiety and depression in the MS patients, our results were in line with those of the above study. Dehghani et al. (2013) found 52.8% of patients had moderate stress, which was in agreement with the findings of the present study. MS as a chronic disease can cause anxiety, stress, and depression due to the sudden occurrence, onset in the youth period, lack of good prognosis, and lack of definitive treatment [17].

In the present study, the patients aged 30–40 years had the highest level of depression, anxiety, and stress. MS occurs mostly in the age range 20–40 years, but the mean age for diagnosis of the disease is 30 [3]. Anxiety, stress, and depression are the main complications of MS so that this disease can negatively affect the quality of life of the patients, cause job loss, family conflicts, and increased divorce, and make the patients susceptible to different mental illnesses such as anxiety, depression, and stress.

In the current study, the patients with good economic status had the lowest level of depression, anxiety, and stress, which was in line with the results of the study by Pham et al. (2018) [10]. Given the high cost of MS treatment in Iran, those with good economic status are less probable to be concerned about the treatment costs. Hence, they seem to have less anxiety, stress, and depression associated with concern about receiving pharmaceutical treatments.

The results of the present study showed stress, anxiety, and depression were higher in retired patients. Esmaili et al. (2009) reported higher stress in unemployed patients [21], which was in agreement with the results of the current study. The patients with primary and junior high school education had the highest level of depression, anxiety, and stress. Giordano et al. (2011) also found the highest level of depression in patients with a lower level of education [7].

In our study, the highest levels of depression, stress, and anxiety were observed in married patients, which is in line with the results of Alsadi et al.(2015) [20]. However, Barzegar et al. (2017) found no significant association between marital status and depression among MS patients in Iran [22]. It seems that married patients undergo more mental stress than single patients due to impaired family responsibilities, which manifests as depression, anxiety, and stress.

Our findings indicated depression and stress were higher in women than in men. The results of the study of Marrie et al. (2018) in British Columbia showed the prevalence of MS was higher in women than in men and women experienced a higher level of depression [23]. The study of Esmaili et al. (2009) in Iran indicated a higher level of stress in women than in men [21], confirming the results of our study. Given the high prevalence of MS in women, the high prevalence of stress, anxiety, and depression among them is not far-fetched.

The mean scores of depression and anxiety were higher in the patients with a family history of MS than those without a family history of MS, which is in agreement with the results of Schiffer et al.(1988) [24]. The results of our study indicated that only the variable job (being an employee) could predict depression despite the significant relationship between depression and education, economic status, and job variables. It should be noted that the focus of Schaffer et al.’s study was to find the relationship between genetic causes and affective symptoms in MS patients. But in the current study, the researchers aimed to determine the relationship between demographic variables and the frequency of depression, stress and anxiety symptoms among MS patients. In another study, Salehpoor et al. (2018) showed limitations due to physical problems, fatigue, and physical pain could significantly predict the depression associated with MS [25]. In this regard, it should be noted that in the study of Salehpoor et al. (2018), variables including physical problems, fatigue, and physical pain were entered into the regression model to predict depression, but in the present study, only occupational and income level variables were included. The present study had several limitations. Firstly, data were collected by self-report, which might have affected the validity of the results. In this regard, it should be noted that patients may not complete the questionnaires carefully, which may lead to measurement bias. Secondly, the present study was cross-sectional, so it was not possible to determine the cause and effect relationship between the variables. Thirdly, the possibility of type 1 error could decrease the validity of the results. Fourthly, the low sample size in the current study could limit the precision.

## Conclusion

MS influences all aspects of the life of an individual, including physical and psychological health. In our study, 47.1% of patients suffered from moderate depression, 39.1% had moderate anxiety, and 44.8% had moderate stress. There was a statistically significant relationship between depression and job, education, and economic status variables and between anxiety and economic status. There was also a statistically significant association between stress and demographic variables. Considering the increasing prevalence of MS, depression, stress, and anxiety levels are suggested to be measured in the MS patients from other regions. Moreover, the effects of interventional measures on depression, anxiety, and stress among these patients are advised to be investigated. In this study, due to racial diversity in Kermanshah, it was not possible to determine the frequency of different races, so it is recommended to determine them in future studies. Furthermore, the government and non-governmental societies are recommended to provide favorable grounds for the employment of these patients. Patients with MS need to be periodically evaluated by psychologists/psychiatrists and treated for symptoms of stress, anxiety, and depression. Nurses can also play a role by listening to patients talk, answering questions, and referring patients with symptoms of stress, anxiety, and depression to a psychiatrist/psychologist.

## Data Availability

Data are available by contacting to the corresponding author.

## Abbreviations

MS: Multiple Sclerosis
DASS-21: Depression Anxiety Stress Scales
KMUS: Kermanshah University of Medical Sciences
